# Dysbiosis in the oral bacterial and fungal microbiome of HIV-infected subjects is associated with clinical and immunologic variables of HIV infection

**DOI:** 10.1371/journal.pone.0200285

**Published:** 2018-07-11

**Authors:** Pranab K. Mukherjee, Jyotsna Chandra, Mauricio Retuerto, Curtis Tatsuoka, Mahmoud A. Ghannoum, Grace A. McComsey

**Affiliations:** 1 Center for Medical Mycology, Department of Dermatology, Case Western Reserve University, Cleveland, Ohio, United States of America; 2 Department of Neurology and Population and Quantitative Health Sciences, Case Western Reserve University, Cleveland, Ohio, United States of America; 3 Infectious Diseases, University Hospitals Cleveland Medical Center and Case Western Reserve University, Cleveland, Ohio, United States of America; Wadsworth Center, UNITED STATES

## Abstract

**Background:**

The effect of smoking on microbial dysbiosis and the potential consequence of such shift on markers of HIV disease is unknown. Here we assessed the relationship of microbial dysbiosis with smoking and markers of HIV disease.

**Methods:**

Oral wash was collected from: (1) HIV-infected smokers (HIV-SM, n = 48), (2) HIV-infected non-smokers (HIV-NS, n = 24), or (3) HIV-uninfected smokers (UI-SM, n = 24). Microbial DNA was extracted and their bacterial and fungal microbiota (bacteriome and mycobiome, respectively) were characterized using Ion-Torrent sequencing platform. Sequencing data were compared using clustering, diversity, abundance and inter-kingdom correlations analyses.

**Results:**

Bacteriome was more widely dispersed than mycobiome, there was no noticeable difference in clustering between groups. Richness of oral bacteriome in HIV-SM was significantly lower than that of UI-SM (*P* ≤ .03). Diversity of HIV-NS was significantly lower than that of HIV-SM or UI-SM at phylum level (*P* ≤ .02). Abundance of Phylum Firmicutes was significantly decreased in HIV-NS compared to HIV-SM and UI-SM (*P* = .007 and .027, respectively), while abundance of Proteobacteria was significantly increased in HIV-NS compared to HIV-SM and UI-SM (*P* = .0005 and .011, respectively). Fungal phyla did not differ significantly between the three cohorts. Cumulative smoking was positively correlated with *Facklamia* but negatively with *Enhydrobacter*, and current alcohol use was negatively correlated with *Geniculata*. Bacteria *Facklamia* exhibited weakly positive correlation with longer PI duration (r = 0.094, *P* = 0.012), and a negative correlation with nadir CD4 count (r = -0.345; *P* = 0.004), while *Granulicatella* was negatively correlated with nadir CD4 count (r = -0.329; *P* = 0.007). Fungus *Stemphylium* correlated negatively with nadir CD4 (r = -0.323; *P* = 0.008).

**Conclusions:**

Dysbiosis of the oral microbiota is associated with clinical and immunologic variables in HIV-infected patients.

## Introduction

HIV infection has been associated with dysbiosis of oral microbiome, with increased levels of pathogenic bacteria and fungi [1, 2], but the effect of smoking on microbiome shift and the consequence of such shift on HIV disease markers is unknown. Bacterial microbiome of healthy individuals has been characterized [3, 4], and our group was the first to characterize the oral mycobiome of healthy individuals [5]. Previous studies have suggested that nonculturable bacteria may be associated with oral diseases [6, 7], obesity [8], gastrointestinal cancers [9], and CVD [10, 11].

The effect of smoking on the composition and pro-inflammatory characteristics of the oral flora in the healthy population has been recently investigated [12–15], with studies suggesting that smoking favors colonization of pathogens like *Fusobacterium* and *Porphyromonas* in oral biofilms, and a concomitant pro-inflammatory response. In the general population, only two smoking cessation studies (n = 11 and 15) are available, showing a decrease in the prevalence of some pathogenic bacteria like *Porphyromonas*, and an increase in *Veillonella*, the latter being favorable for periodontal health as it inhibits colonization by pathogenic pathogens [12, 13]. No alteration in the microbiome was found following periodontal therapy in those who continued to smoke [12], suggesting the changes observed were the result of smoking cessation. These studies did not assess fungal species, nor the effect of the change in oral microbiome on markers of HIV infection. Here we present the first comprehensive study that assessed the effect of smoking on oral bacterial and fungal microbiome in HIV-infected subjects, and the relationship between oral microbiome and markers of HIV infection (higher viral load, nadir and current CD4 level) and cardiovascular risk.

## Results

### Sample demographics

Oral wash samples were collected from HIV-infected (n = 72) and uninfected individuals (n = 24). The HIV-positive group included 48 smokers and 24 non-smokers, while all 24 uninfected individuals were active smokers (Table 1).

**Table 1:** Study demographics*.

Variable	HIV-infected Non-smoker (HIV-NS)	HIV-infected Smoker (HIV-SM)	Uninfected Smoker (UI-SM)	P^¶^
N	24	48	24	
Age (years, mean ± SD)	44.99 ± 11.98	46.44 ± 10.56	42.74 ± 13.77	.458
BMI	28.54 ± 4.03	25.96 ± 5.49	28.29 ± 4.92	.010^§^
CD4, Current	808.83 ± 382.58	838.87 ± 344.85	-	.564
CD4, Nadir	240.3 ± 206.76	189.46 ± 189.65	-	.202
Pack Years	-	24.68 ± 17.1	21.58 ± 14.52	.000^†^
ARV Duration (Months)	135.62 ± 77.83	118.2 ± 75.26	-	.444
Gender (N, %)				.075^‡^
Female	3 (12.5%)	8 (16.6%)	9 (37.5%)	
Male	21 (87.5%)	40 (83.3%)	15 (62.5%)	
Race (N, %)				.704^‡^
African American	15 (62.5%)	35 (72.9%)	17 (70.8%)	
Caucasian	8 (33.3%)	10 (20.8%)	7 (29.2%)	
Hispanic	1 (4.1%)	2 (4.1%)	0 (0%)	
Other	0 (0%)	1 (2.1%)	0 (0%)	
Percent on PI (N, %)				.660^‡^
No	13 (54.2%)	27 (56.2%)	-	
Yes	11 (45.8%)	21 (43.7%)	-	

*For numerical variables (Age, BMI, Current CD4, Nadir CD4, Pack years, and ARV duration) cell entries are sample mean ± standard deviation.

^¶^P-values for numerical variables are based on Kruskal-Wallis test across all groups. For pairwise comparisons, P-values are based on Wilcoxon test, adjusted for multiple comparisons (Holm)

^§^P = .014 for HIV-SM vs. HIV-NS

^†^P < .001 for HIV-SM vs. HIV-NS or UI-SM. P > .05 for all other pairwise comparisons. For categorical variables (Race, Gender and Percent on PI), percentages in cell entries are within group, with Race categories combined to African American versus non-African American.

^‡^P-values for categorical variables are based on two-sided Fisher’s exact test.

### Clustering analyses

**Fig 1:**
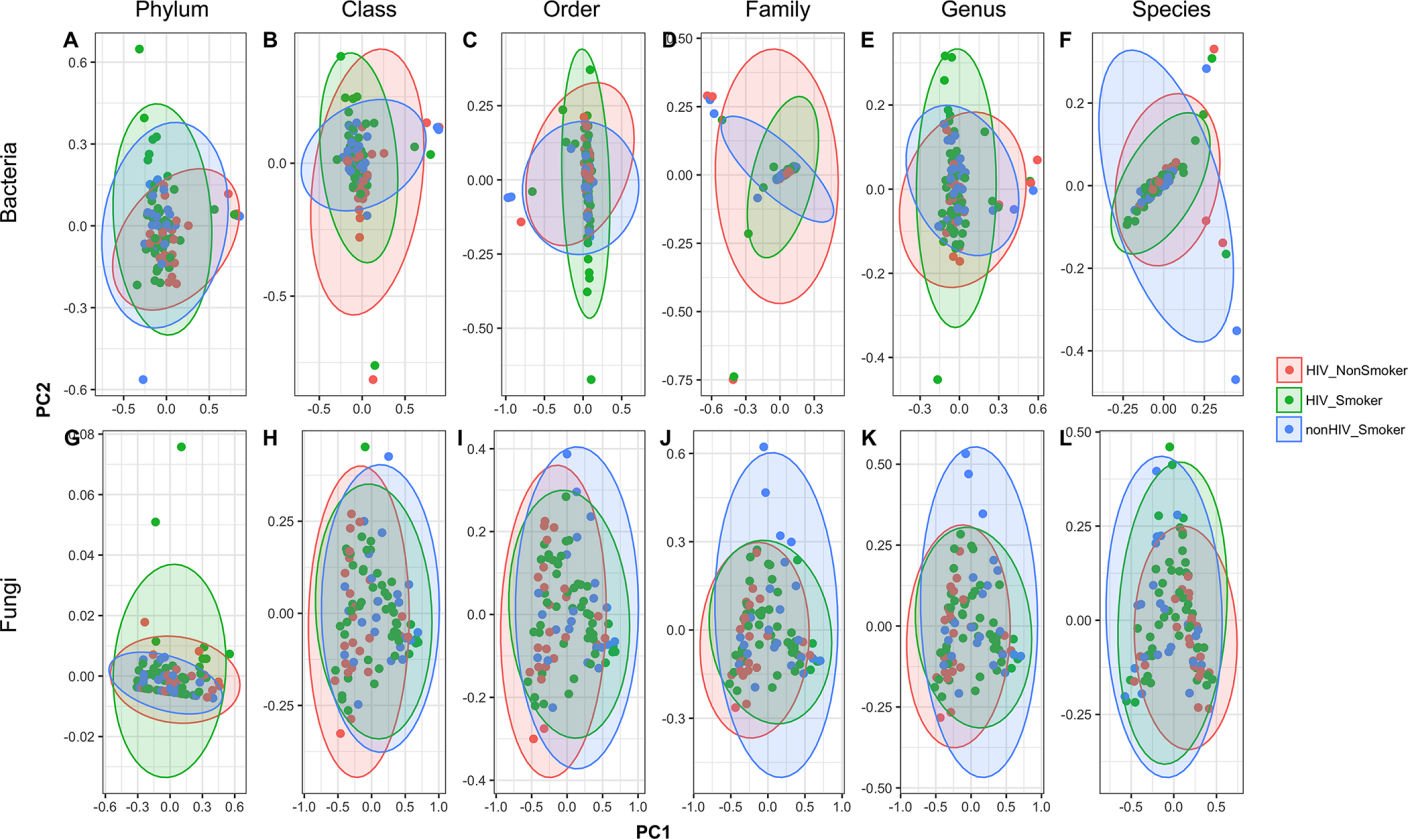
**Principal coordinates analysis (PCoA) of (A-F) bacteriome and (G-L) mycobiome data at different taxa in the three groups.** Confidence ellipses are shown for each group, at 0.95% confidence.

Principal coordinate analysis (PCoA) showed that while the bacteriome data were more widely dispersed than the mycobiome, there was no noticeable difference in clustering between the HIV-infected smokers, HIV-infected non-smokers, and uninfected smokers groups (Fig 1). Moreover, 99% variation was explained by three variables in bacteriome (Proteobacteria, Firmicutes and Bacteroidetes) and one variable in the mycobiome (Ascomycota), for all three groups (data not shown). We found that in the mycobiome, the uninfected smoker group exhibited significantly different clustering (based on distance to centroid) compared to both HIV-infected groups at Family and Genus levels (panels J,K in S1 Fig, P ≤ .018). At the species level of mycobiome, this difference was detected only between uninfected smokers and HIV-infected smokers (panel L in S1 Fig, P = .037).

### Diversity and richness analyses

**Fig 2:**
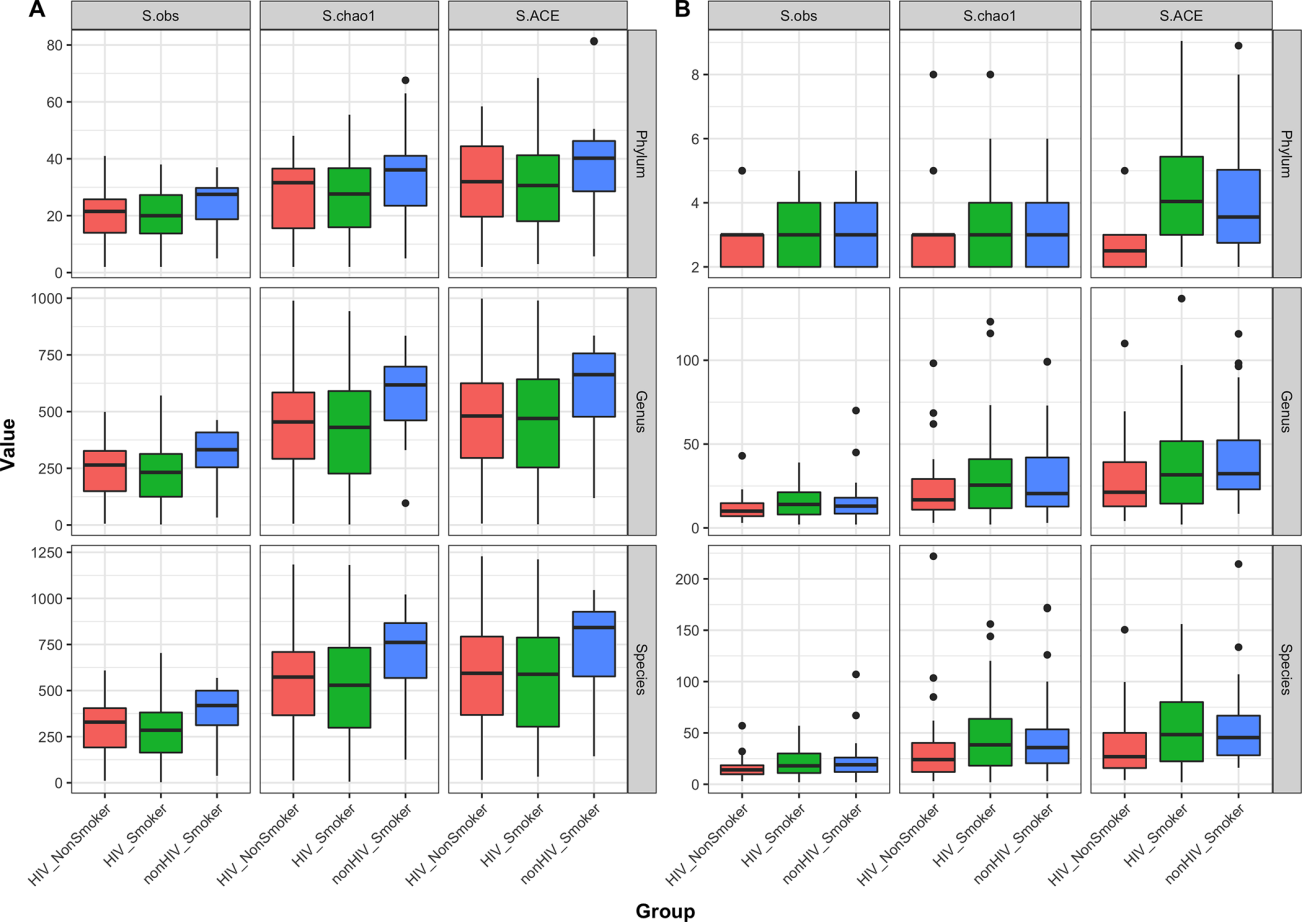
Boxplots showing richness estimates (observed, chao1 and ACE) of (A) bacteriome and (B) mycobiome at phylum, genus, and species levels.

Shannon diversity index of bacteriome did not differ significantly between the smoker and non-smoker groups of HIV-infected patients (data not shown), while richness of oral bacteriome in HIV-infected smokers was significantly lower than that of uninfected smokers (compared using Chao1 and ACE measures, *P* ≤ .03, Fig 2A). Diversity of mycobiome in HIV-infected non-smokers was significantly lower than that of HIV-infected or uninfected smokers at phylum level (*P* ≤ .02), but not at genus level. There was no difference in richness of the mycobiome at any taxon level (Fig 2B).

### Core bacteriome and mycobiome

**Fig 3:**
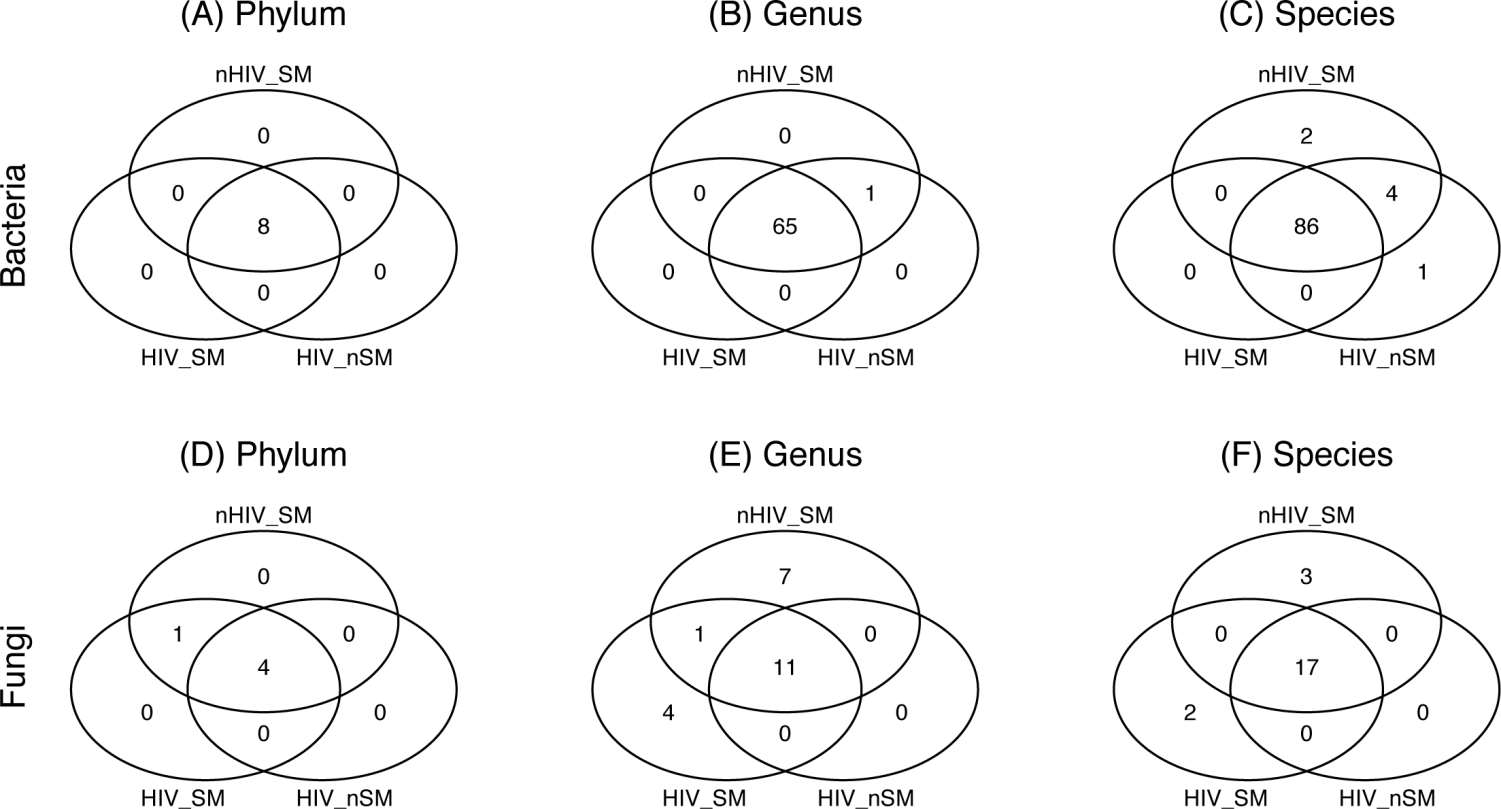
**Venn diagrams showing frequency distribution of (A-C) core bacterial and (D-F) core fungal taxa (detected at abundance > 1%) in the three study groups.** Frequency distribution in the core microbiota are shown for (A,D) Phylum, (B,E) Genus and (C,F) Species levels. HIV-SM: HIV-infected smokers, HIV-nSM: HIV-infected non-smokers, nHIV-SM: uninfected smokers.

The core bacteriome (comprising taxa detected with a frequency of at least 20%, at an abundance > 1%) comprised eight bacterial phyla, 65 genera, and 86 species common in all three study groups (Fig 3A–3C). Genus *Pelomonas* was unique to HIV-infected non-smoker and uninfected smoker groups (Fig 3B). Bacterial species *Pseudomonas pseudoalcaligenes* was detected only in HIV-infected non-smokers, while two *Pelomonas* species were present in only the uninfected smoker group (*Pelomonas puraquae* and *Pelomonas* sp., Fig 3C).

Analysis of core mycobiome revealed four fungal phyla were common to all three groups, and one (Chytridiomycota) was unique to the HIV-infected and uninfected smoker groups (Fig 3D). At the genus level, 11 fungal genera were common to all cores in the three groups, 7 were unique to uninfected smoker group, while 4 (*Cladosporium*, *Nakaseomyces*, *Scleroderma*, and *Rhodotorula*) were detected only in the HIV-infected smoker group (Fig 3E). At the species level, the mycobiome showed 17 species to be common to all three groups, two species (*Candida glabrata* and *Scleroderma* sp) were unique to HIV-infected smoker group, while three species (*Stemphylium solani*, *Debaryomyces hansenii*, and *Olpidium brassicae*) were detected only in the uninfected smoker group (Fig 3F).

### Abundance profiles

**Fig 4:**
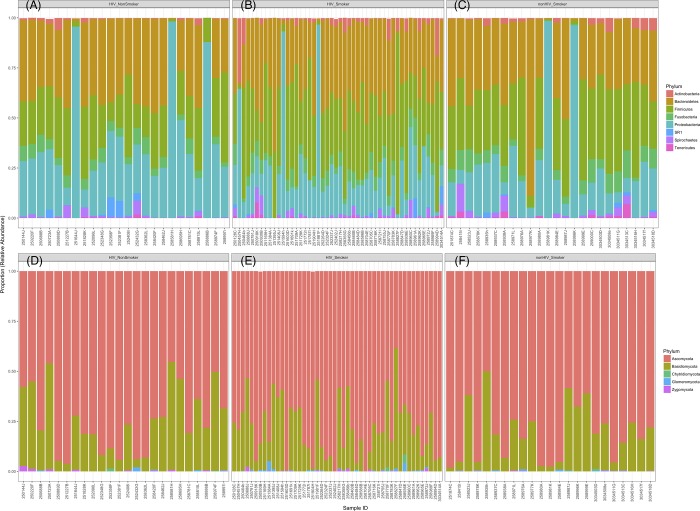
**Stacked bar charts showing distribution of (A-C) bacterial and (D-F) fungal phyla across the tested samples in the three groups.** Phyla present at an abundance of at least 1% relative to the total abundance in each sample were included in the analyses.

**Fig 5:**
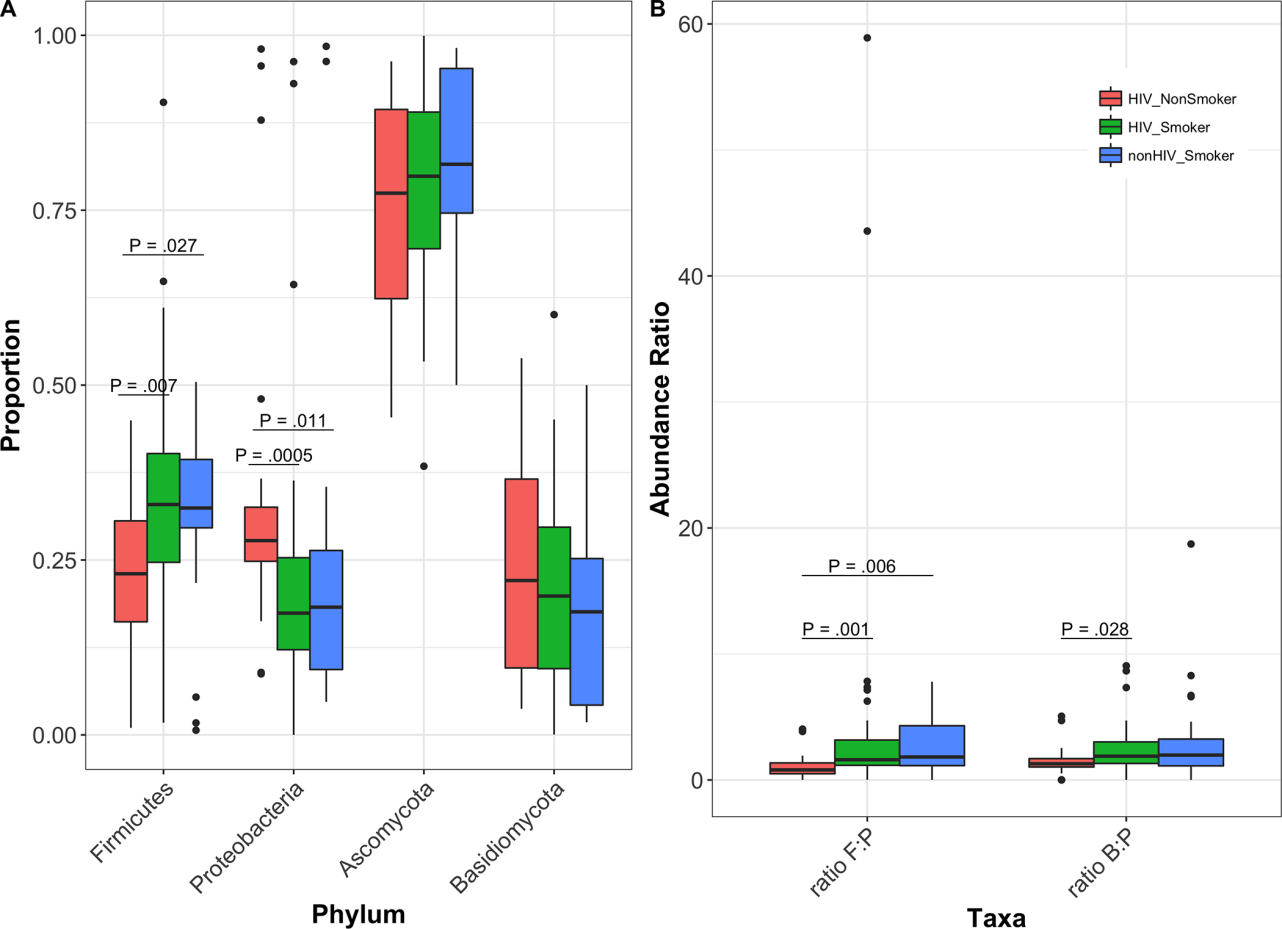
Abundance profile of bacterial and fungal phyla in study groups. (A) Boxplots showing relative abundance of bacterial and fungal phyla, (B) abundance ratio of Fusobacteria:Proteobacteria (ratio F:P) and Bacteriodetes:Proteobacteria (ratio B:P).

Most abundant phyla in HIV-infected smokers were Firmicutes > Bacteroidetes > Proteobacteria, while in HIV-infected non-smokers were Proteobacteria > Bacteroidetes > Firmicutes (Fig 4). The distribution of phyla in the non-HIV infected smokers were Bacteroidetes > Firmicutes > Proteobacteria. In the mycobiome, Ascomycota followed by Basidiomycota were the most abundant phyla for all three groups (Fig 4). Abundance of Firmicutes was significantly decreased in HIV-infected nonsmokers compared to HIV-infected smokers and uninfected smokers (*P* = .007 and .027, respectively, Fig 5A). In contrast, abundance of Proteobacteria was significantly increased in HIV-infected nonsmokers compared to HIV-infected smokers and uninfected smokers (*P* = .0005 and .011, respectively, Fig 5A). Fungal phyla did not differ significantly between the three cohort groups (Fig 5A). Comparisons of bacterial genera showed that levels of *Granulicatella*, *Lactobacillus*, *Veillonella*, and *Enhydrobacter* were significantly increased, while that of *Neisseria* were significantly decreased, in HIV-infected smokers compared to infected non-smokers (Table 2). Levels of four fungal genera were significantly different between the three cohorts in the study (Table 3), including an unidentified Myxotrichaceae, *Stemphylium*, *Candida*, and *Chroogomphus*. Interestingly, levels of *Candida* were increased in both HIV-infected and uninfected smoker groups (34.7% and 38.4%, respectively) compared to the HIV-infected non-smoker group (19.2%, *P* = 0.12 and 0.04, respectively). Our results also revealed a non-significant increase in levels of *C*. *dubliniensis* in the HIV-infected smokers (7.3%) compared to HIV-infected non-smokers (1.8%, *P* = 0.1; Table 4). In addition, *C*. *rugosa* was detected only in the uninfected smoker group (0.2%). We also found that ratio of the abundance of Fusobacteria:Proteobacteria (F:P) was significantly decreased in HIV-infected non-smokers compared to both smoker groups (P ≤ .006), while the abundance ratio of Bacteriodetes:Proteobacteria (B:P) was significantly decreased in HIV-infected non-smokers compared to HIV-infected smoker group (P = .028, Fig 5B).

**Table 2:** Bacterial genera with significantly different abundance between the three groups.

Bacterial Genus	HIV-infected Non-Smoker (A)	HIV-infected Smoker (B)	Uninfected Smoker (C)	*P* (A vs B)	*P* (A vs C)	*P* (B vs C)
Prevotella	22.1%	23.8%	19.1%	1.000	0.255	0.175
Streptococcus	16.2%	20.6%	19.6%	0.430	0.551	1.000
Haemophilus	10.0%	8.1%	7.9%	0.855	1.000	1.000
Neisseria	7.0%	2.8%	2.8%	0.012	0.012	1.000
Granulicatella	1.5%	2.6%	2.4%	0.033	0.186	1.000
Lactobacillus	0.4%	2.7%	2.7%	0.009	0.019	1.000
Parvimonas	0.3%	0.3%	0.7%	1.000	0.068	0.014
Veillonella	0.2%	0.7%	0.3%	0.010	0.823	0.140
Enhydrobacter	0.1%	0.1%	5.0%	0.003	1.000	0.050
Pelomonas	0.1%	0.2%	0.0%	0.445	1.000	0.036
Stenotrophomonas	0.0%	1.6%	0.0%	0.290	1.000	0.048
Facklamia	0.0%	0.0%	0.1%	0.205	0.002	0.155

**Table 3:** Fungal genera with significantly different abundance between the three groups.

Fungal Genus	HIV-infected Non-Smoker (A)	HIV-infected Smoker (B)	Uninfected Smoker (C)	*P* (A vs B)	*P* (A vs C)	*P* (B vs C)
Unidentified Myxotrichaceae	47.2%	33.1%	25.6%	0.022	0.005	0.281
Candida	19.2%	34.7%	38.4%	0.118	0.044	1.000
Chroogomphus	18.2%	12.9%	9.6%	0.361	0.024	0.318
Saccharomyces	1.7%	1.1%	3.7%	1.000	1.000	1.000
Stemphylium	0.4%	0.0%	0.1%	1.000	0.782	0.040

**Table 4:** Abundance of *Candida* species in the tested samples.

*Candida* species	HIV-infected Non-Smoker (A)	HIV-infected Smoker (B)	Uninfected Smoker (C)	P-value (A vs. B)	P-value (A vs. C)	P-value (B vs. C)
*Candida albicans*	6.8%	11.8%	14.5%	1.000	0.310	0.896
*Candida dubliniensis*	1.8%	7.3%	5.1%	0.105	0.346	1.000
*Candida ethanolica*	0.0%	0.0%	0.1%	NA	0.655	0.314
*Candida orthopsilosis*	0.1%	0.2%	0.3%	1.000	0.144	0.483
*Candida oxycetoniae*	0.0%	0.1%	0.0%	1.000	1.000	1.000
*Candida parapsilosis*	0.2%	0.1%	0.4%	1.000	0.557	0.375
*Candida rugosa*	0.0%	0.0%	0.2%	NA	0.306	0.083
*Candida sp*.	8.5%	11.2%	16.1%	1.000	0.170	0.259
*Candida sp 3 2*	0.0%	0.0%	0.1%	NA	0.655	0.314
*Candida sp HN 26*	0.0%	0.1%	0.0%	1.000	1.000	1.000
*Candida sp L2683B*	0.0%	0.0%	0.1%	NA	0.655	0.314
*Candida tropicalis*	1.5%	3.7%	1.4%	1.000	1.000	1.000
*Candida versatilis*	0.0%	0.0%	0.0%	0.996	NA	1.000
*Candida lusitaniae*	0.0%	0.1%	0.0%	0.157	0.110	1.000

### Intra- and inter-kingdom correlations

**Fig 6:**
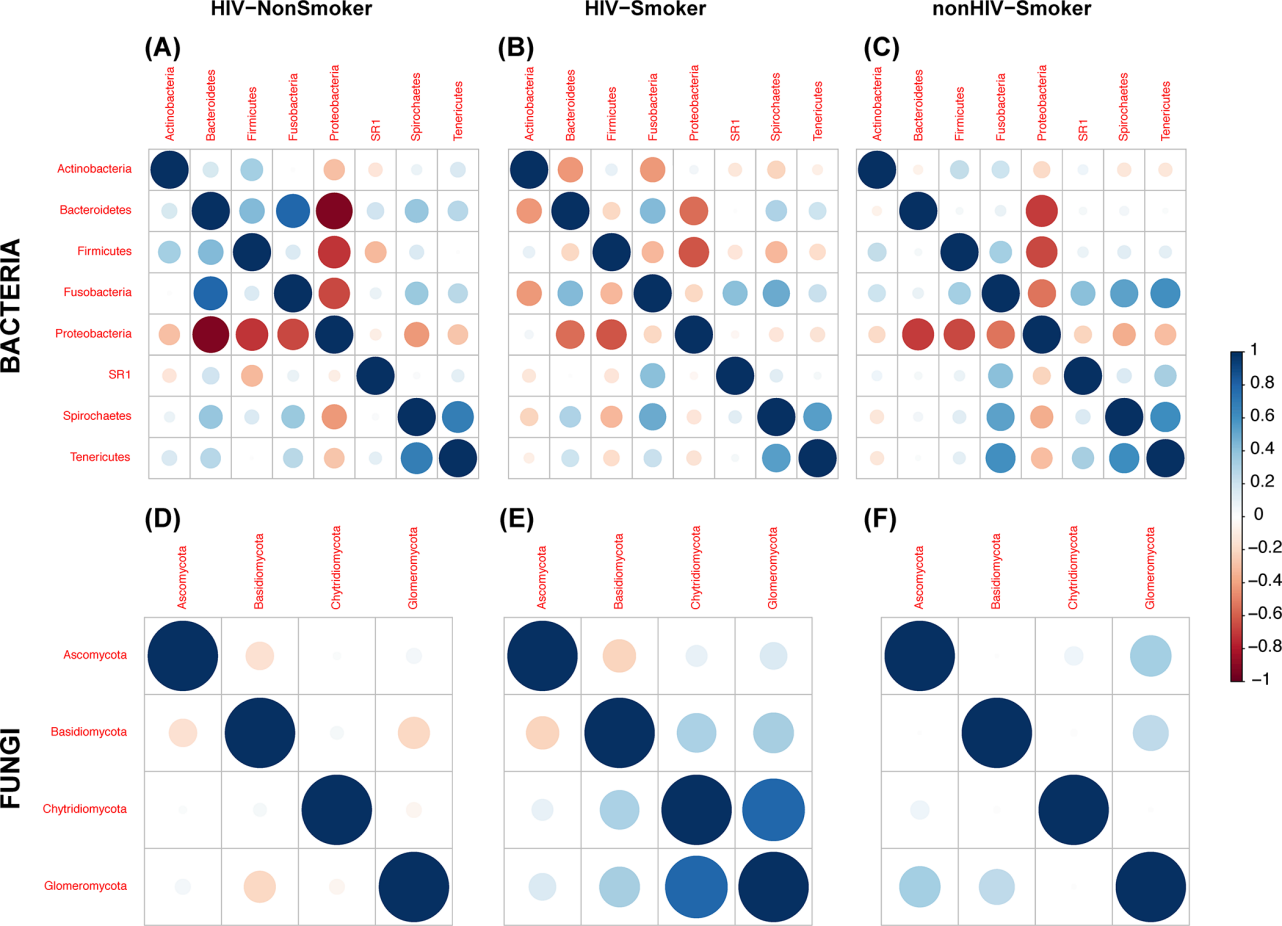
**Intra-kingdom correlations within the bacteriome and mycobiome for (A,D) HIV-infected non-smokers, (B,E) HIV-infected smokers, and (C,F) uninfected smokers.** Spearman’s correlation for each comparison was determined for the three groups. Blue circles indicate positive correlations; red circles indicate negative correlation; diameter of circles represent the absolute value of correlation for each pair of the microbe-microbe matrix.

**Fig 7:**
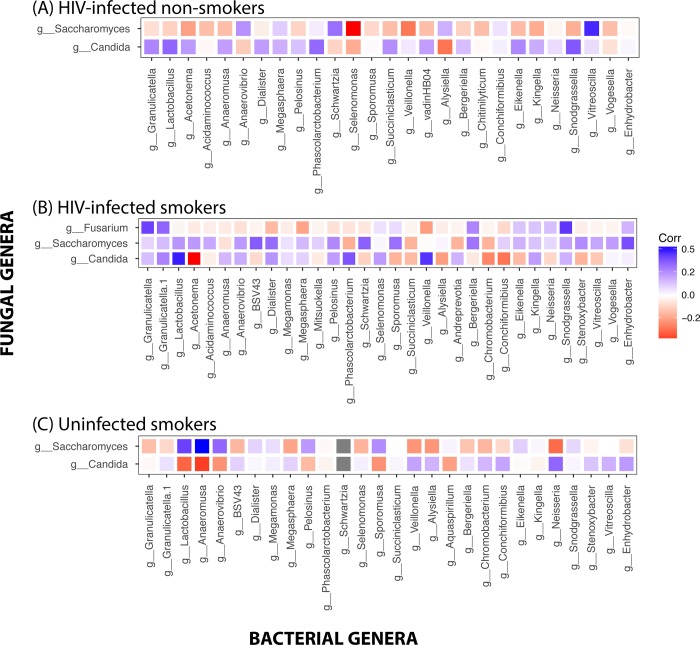
**Inter-kingdom correlations between the bacteriome and mycobiome for (A) HIV-infected non-smokers, (B) HIV-infected smokers, and (C) uninfected smokers.** Spearman’s correlation for each comparison was determined for the three groups. Blue tiles indicate positive correlation; red tiles indicate negative correlations; tile sizes represent the absolute value of correlation for each pair of the microbe-microbe matrix.

Next, we determined the significant intra- and inter-kingdom correlations in the bacteriome and mycobiome at phylum and genus levels. Correlations of Actinobacteria with other phyla differed between the three cohort groups, where negative correlations were detected with Bacteroidetes and Fusobacteria in HIV-infected smoker group, while positive correlations were noted in the other two groups (Fig 6A–6C). In the mycobiome, correlations of Ascomycota and Basidiomycota with the bacterial phyla exhibited distinct patterns in the three study groups (Fig 6D–6F). Analyses of inter-kingdom interactions revealed that fungal Phylum Glomeromycota exhibited significant positive correlation with bacterial Phylum Tenericutes (r = 0.88, *P* < .001) in HIV-infected non-smokers. We also explored the inter-kingdom interactions of fungal and bacterial genera that were significantly different between the groups. Abundance of *Candida* was correlated negatively with *Neisseria* in HIV-infected smokers, positively in infected non-smokers, and uninfected smokers (Fig 7). Interestingly, *Candida* was positively correlated with *Lactobacillus* in HIV-infected smokers and non-smokers, but negatively correlated in uninfected smokers. In contrast, *Saccharomyces* was negatively correlated with Neisseria in uninfected smokers, but positively correlated in both HIV-infected (smoker and non-smoker) groups. However, these inter-kingdom correlations of *Candida* and *Saccharomyces* did not reach statistical significance.

### Correlations between microbial genera and clinical variables

Next, we determined the correlations between microbial (bacterial and fungal) genera and clinical variables. *Facklamia* correlated positively with age (r = 0.209, *P* = 0.048) and cumulative smoking (in pack-years), (r = 0.303, *P* = 0.004), while *Enhydrobacter* exhibited negative correlation with pack-years (r = -0.241, *P* = 0.024). *Geniculata* was negatively correlated with current alcohol use (r = -.267, *P* = 0.046). No significant correlations were observed between any bacteria and BMI. Several bacteria correlated with HIV-immunologic parameters markers and HIV treatment factors; *Facklamia* also exhibited weakly positive correlation with longer PI duration (r = 0.094, *P* = 0.012), and a negative correlation with nadir CD4 count (r = -0.345; *P* = 0.004). *Granulicatella* also was negatively correlated with nadir CD4 count (r = -0.329; *P* = 0.007). No bacteria correlated with current CD4 counts, or ART or NRTI duration. Several fungi correlated with markers of HIV-clinical and immunologic parameters, with *Stemphylium* correlated negatively with nadir CD4 (r = -0.323; *P* = 0.008). No fungi correlated significantly with current alcohol use, cumulative smoking, age, BMI, current CD4 cell count, or ART or HIV duration.

## Discussion

To our knowledge, our study is the first to comprehensively characterize differences in the oral bacteriome and mycobiome concomitantly in HIV-infected smokers and non-smokers. We found that while the within-group variation in microbiota was similar between the three groups, fungal diversity of the mycobiome was significantly increased in HIV-infected smokers compared to non-smokers, suggesting that smoking induces changes in the oral mycobiome. Previous investigations have also shown that oral bacteriome varies between smokers and non-smokers. One such study conducted by the Lung HIV Microbiome Project (LHMP), Morris et al [16] focused on healthy individuals, and compared the bacterial microbiome of oral washes and bronchoscopic alveolar lavages (BAL) (representing the upper and lower respiratory tract, respectively) in healthy nonsmokers and smokers (n = 64). These investigators reported that majority of the bacteria were common between the mouth and the lung and that levels of *Porphyromonas*, *Neisseria*, and *Gemella* in the oral communities were significantly decreased in smokers compared to nonsmokers, which is in agreement with our finding that abundance of *Neisseria* was significantly decreased in HIV-infected smokers compared to infected non-smokers. A subsequent study by the same group comparing the oral and lung bacteriomes of uninfected and HIV-infected individuals [17] reported that differences in microbial populations were detected only in the oral washes among the subject groups (Streptococcus, Actinomyces, Rothia, and Atopobium), but not in the lower respiratory tract (BAL). Our results are in agreement with these findings, since we also found that abundance of specific bacterial taxa (*Granulicatella*, *Lactobacillus*, *Veillonella*, *Enhydrobacter*, Streptococcaceae and Comamonadaceae) were significantly increased in HIV-infected smokers compared to infected non-smokers. In addition, abundance of the fungal genus *Candida* was increased in HIV-infected smokers. While previous studies characterized the oral bacteriome in healthy smokers or HIV-infected patients (compared to healthy non-smokers or uninfected healthy individuals, respectively) our results provide insight about dysbiosis of bacteriome and mycobiome concomitantly in HIV-infected smokers and non-smokers, as well as uninfected smokers. Inter-kingdom correlations between two known pathogenic genera (*Candida* and *Neisseria*) were negative in HIV-infected smokers but positive in infected non-smokers. While this correlation did not reach statistical significance, it raises the possibility that the two genera may interact and influence microbial dysbiosis in the oral cavity.

Cumulative smoking was positively correlated with *Facklamia* and negatively correlated with *Enhydrobacter*, but did not correlate with *Fusobacterium* and *Porphyromonas*, as was seen in studies of non-HIV infected populations. Additionally, both bacteria (*Facklamia* and *Granulicatella*) and fungi (*Chroogomphus*, *Microbotryum*, *Stemphylium*) correlated with worse HIV immunologic parameters, higher viral load and more advanced disease (i.e. lower nadir CD4). Consumption of alcohol did not correlate positively with any bacteria or fungi, which was unexpected negative finding that is worth investigating further. Our findings are notable for the fact that we identified fungi other than *Candida* (the most common fungal colonizer in HIV infection), to be correlated with HIV disease markers. The clinical relevance of these correlations remains to be explored.

One potential caveat of the current study is that the results are derived based on mouthwash samples. While oral wash collection is non-invasive and relatively simple to implement, it does not account for the high diversity in local bacterial niches in the oral cavity, including biofilms, gingival, plaque. Despite this limitation, our results provide a global snapshot of the oral microbiome and may reveal novel linkages between the microbiota and HIV infection and its covariates. Samples were collected at a single time point in the current study, therefore, it was not possible to determine whether changes in microbiome were related to progression of disease or response to therapy. In addition, since the intra-kingdom correlations are based on compositional data, these correlations need to be validated in larger cohort studies and targeted functional assays.

Smoking is associated with periodontal disease, which in turn is also related to HIV infection, and could be a confounder in some of our findings. However, none of the patients in the current study had any clinical symptoms of periodontal disease (redness, swelling or bleeding). However, a formal exam performed by a dentist to rule out mild periodontal disease was not part of the clinical protocol.

The clinical relevance of these findings is unknown, and the consequences of such changes, including on cardiovascular disease risk and systemic inflammation, need to be investigated in future studies.

## Materials and methods

### Study population

The study was approved by the IRB of University Hospital Cleveland Medical Center, and all participants signed a written informed consent before any evaluation was performed. Participants were recruited for this study and by design assigned to three different groups as follows: (1) HIV-infected smokers (HIV-SM), (2) HIV-infected non-smokers (HIV-NS), and (3) HIV-uninfected smokers (nonHIV-SM).

### Inclusion criteria

**(1) For HIV-infected smokers (n = 48):** men and women 18 years of age or older were recruited. They were receiving stable Antiretroviral therapy (ART) for at least 12 weeks prior to study entry, with cumulative prior ART of at least 6 months, with plasma HIV-1 RNA level of ≤ 400 copies/ml, and were smoking on average ≥10 cigarettes/day for ≥6 months and had a history of ≥5 cumulative years of smoking.

**(2) For HIV-infected non-smokers (n = 24):** criteria were similar as above, except they were non-Smokers, defined as having never smoked or smoked for less than 3 months cumulative during lifetime and not within the last 12 months.

**(3) For HIV uninfected smokers (n = 24): HIV-uninfected** healthy men and women smokers, 18 years of age or older, with no regular use who currently smoke ≥10 cigarettes/day for ≥6 continuous months and history of ≥5 cumulative years of smoking.

### Exclusion criteria

History of myocardial infarction, stroke, or coronary revascularization procedure, pregnancy/ breastfeeding, Any active or chronic uncontrolled inflammatory condition, Current alcohol or recreational drug use which interferes with the subject’s ability to comply with dosing schedule and protocol evaluations, se of the systemic cancer chemotherapy, or immumodulating agents, uncontrolled thyroid disease or diabetes, liver enzymes > 2.5 x upper limit of normal, Hemoglobin < 9.0 g/dL, or calculated creatinine clearance <50 mL/min. None of the patients had any clinical symptoms of periodontal disease (redness, swelling or bleeding) however we did not have formal exam performed by a dentist to rule out mild periodontal disease.

### Collection of oral wash samples

Oral wash samples were collected from the above three groups as describe earlier[18]. Briefly 20–25 ml saline was emptied into separately labelled blue capped 50-mL Falcon™ centrifuge tubes (Fisher Scientifics Co.). Each subject swished and gargled the saline from the tube in mouth for 2 minutes and expectorated the rinse into the tube. The tubes were closed tightly and stored in -80 till further use. Before use, all the tubes with oral wash samples were completely thawed on ice. Tubes were then centrifuged at 4000 rpm for 15 minutes and 20–25 ml supernatant transferred into fresh 50-mL centrifuge tubes. The pellet left in each tube was used to extract DNA for microbiome studies.

### DNA extraction

Fungal and bacterial genomic DNA was isolated and purified with the QiaAmp DNA Mini Kit (Qiagen) following the manufacturer’s instructions with minor modifications. Briefly, 3 additional bead-beating steps (Sigma-Aldrich beads, diameter = 500 μm) with the MP Fastprep-24 (speed setting of 6, 3 runs of 60s) were performed. The quality and purity of the isolated genomic DNA was confirmed spectrophotometrically using NanoDrop 2000 device (Fisher Scientific SAS, Illkirch, France). DNA concentration was quantified using the Qubit 2.0 instrument applying the Qubit dsDNA HS Assay (Life Technologies, USA). Extracted DNA samples were stored at -20°C.

### Microbiome analyses

Analysis of the microbiome profile in the extracted DNA samples was conducted as described previously by our group [1, 18]. A brief summary of the method is provided below.

#### Amplicon library preparation

The Internal Transcribed Spacer 1 (ITS1) and 16S rDNA regions for fungi and bacteria, respectively, were amplified as described previously [1]. Briefly, ITS1 region was amplified using ITS1F (CTTGGTCATTTAGAGGAAGTAA) and ITS 2 (GCTGCGTTCTTCATCGATGC) primers, and the V4 region of the 16S rRNA gene was amplified using16S-515F: GTGCCAGCMGCCGCGGTAA and 16s-806R: GGACTACHVGGGTWTCTAAT primers. PCR reactions were carried out on 100 ng template DNA, in 50 μl (final volume) reaction mixture consisting of Dream Taq Green PCR Master Mix (ThermoScientific), 0.1g/L bovine serum albumin, 1% of dimethyl sulfoxide (DMSO), 6 mM MgCl_2_, and a final primer concentration of 400nM. PCR conditions were: initial denaturation at 94°C for 3 min, 35 cycles of denaturation for 30s each at 94°C, annealing at 50°C for 30 s, extension at 72°C for 1 min, final extension of 5 min at 72°C. PCR products were sheared for 20 min, using Ion Shear Plus Fragment Library Kit (Life Technologies, NY, USA). Amplicon library was generated with sheared PCR products using Ion plus Fragment Library kits (<350 bp) according to the manufacturer’s instructions, and the library was barcoded with Ion Xpress™ Barcode Adapter, and ligated with the A and P1 adaptors.

#### Sequencing, classification and analysis

The adapted barcoded libraries were equalized using the Ion Library Equalizer kit to a final concentration of 100 pM, pooled and diluted to 26 pM, and attached to the surface of Ion Sphere particles (ISPs) using an Ion PGM Template OT2 200bp kit v2 (Life Technologies, USA) according to the manufacturer's instructions, via emulsion PCR. Quality of ISPs templates was checked using Ion Sphere™ Quality Control Kit (Part no. 4468656) with the Qubit 2.0 device. Sequencing of the pooled libraries was carried out on the Ion Torrent Personal Genome Machine (PGM) system using the Ion Sequencing 200 kit v2 (all from Life Technologies) for 150 cycles (600 flows), with a 318 chip following the manufacturer's instructions. De-multiplexing and classification was performed using the Qiime 1.6 platform. The resulting sequence data were trimmed to remove adapters, barcodes and primers during the de-multiplexing process. In addition, the bioinformatics process filters were applied to the sequence data for the removal of low-quality reads below Q25 Phred score and denoised to exclude sequences with read length below 100 bp[19]. De novo operational taxonomic units (OTUs) were clustered using Uclust algorithm and defined by 97% sequence similarity[20]. Classification at the species level was referenced using the UNITE 5.8s database and taxa assigned using the nBlast method with a 90% confidence cut-off [21, 22].

#### Bioinformatics and statistical analyses

The statistical programming language *R* and related packages [23] were used for diversity and correlation analyses, and Kruskal-Wallis (non-parametric) analysis of variance using abundance data. Principal coordinates analysis (PCoA) was conducted with identified reads/OTUs using classical multidimensional scaling (functions *dist*/euclidean, *cmdscale*) to analyze distribution of dissimilarities and hierarchical clustering in dendrograms in the R package *vegan*. Statistical significance in differences between clustering groups were determined using *adonis/permanova* and *betadisper* functions followed by Tukey's HSD test for pairwise comparisons. Diversity was analyzed using Shannon diversity index (characterizes species diversity) and abundance-based richness estimates (observed, bias-corrected Chao and ACE) were calculated using *vegan* [24]. Taxa with a frequency of at least 20% (and relative abundance >1%) were considered to comprise the core biome in our study. Intra- and inter-kingdom correlations were determined using the *corrplot* package in *R*. For hypothesis tests, *P* < .05 was considered statistically significant, except for pairwise comparisons among groups, where a Bonferroni correction was applied, and *P* < .0167 was considered the significance threshold. All group-wise comparisons were conducted with SPSS (ver. 24). Sequencing data files are available at https://drive.google.com/drive/folders/1aPWhf6DDB6FGaCd8d1lCtXfLCj1g4LEs?usp=sharing.

## Supporting information

S1 Fig Clustering differences, based on distance to centroid, in samples from the three groups at different taxa in bacteria (A-F) and fungi (G-L).
(TIF)
