# Supplementary figures and images for: Dysbiosis in the oral bacterial and fungal microbiome of HIV-infected subjects is associated with clinical and immunologic variables of HIV infection
Source: PLoS One. 2018 Jul 11;13(7):e0200285. doi: 10.1371/journal.pone.0200285 (PMC6040710; doi:10.1371/journal.pone.0200285)

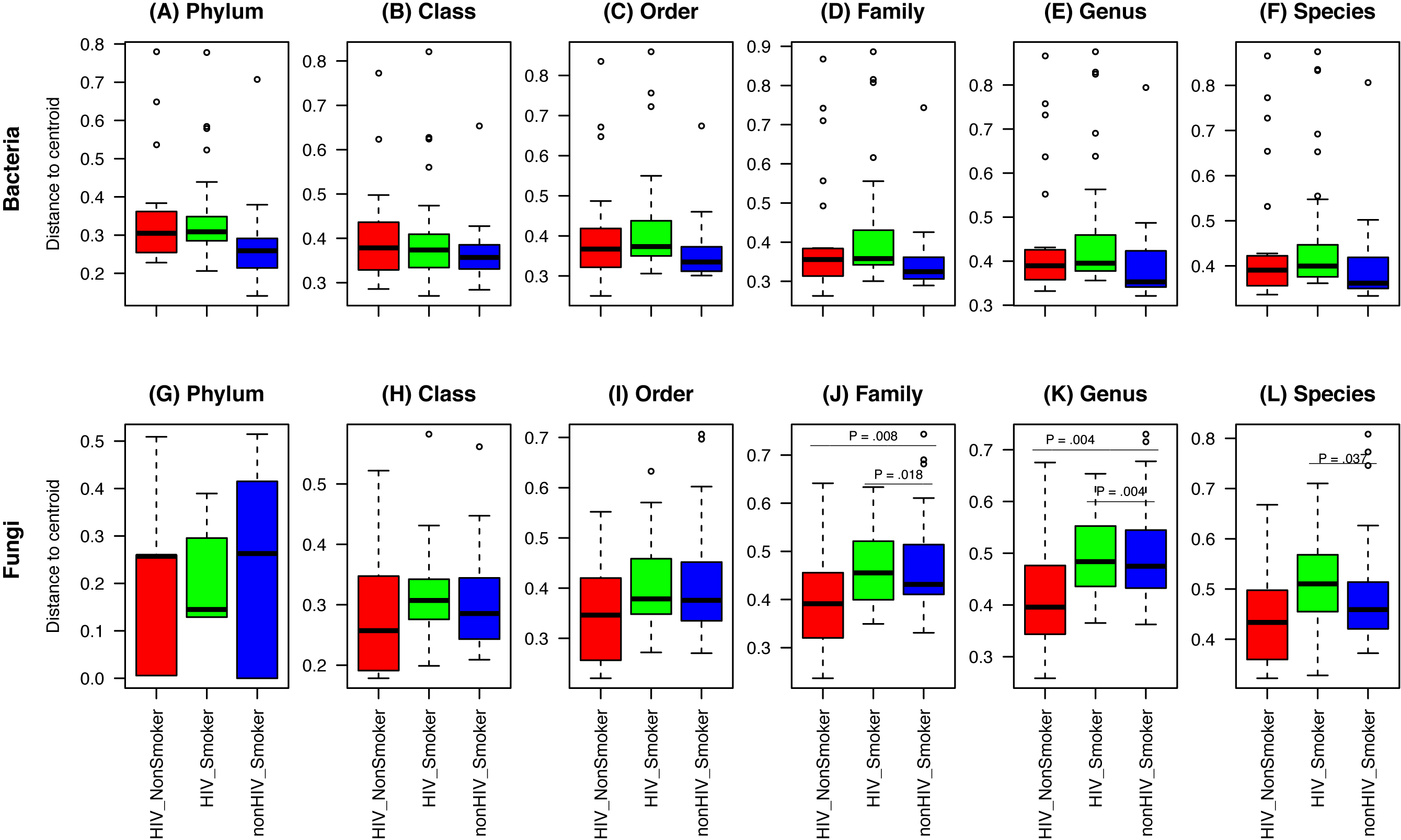

Supplement: S1 Fig — Clustering differences, based on distance to centroid, in samples from the three groups at different taxa in bacteria (A-F) and fungi (G-L). (TIF) [file pone.0200285.s001.tif]
